# Supplementary material for: On the Development of Nanocomposite Covalent Associative Networks Based on Polycaprolactone and Reduced Graphite Oxide
Source: Nanomaterials (Basel). 2022 Oct 25;12(21):3744. doi: 10.3390/nano12213744 (PMC9654163; doi:10.3390/nano12213744)
Supplement: Supplementary file 1 [file nanomaterials-12-03744-s001.zip › nanomaterials-1980908-supplementary.pdf]

Supporting information

On the development of nanocomposite covalent associative networks based on polycaprolactone and reduced graphite oxide

Alberto Vallin, Daniele Battegazzore, Giacomo Damonte, Alberto Fina and Orietta Monticelli

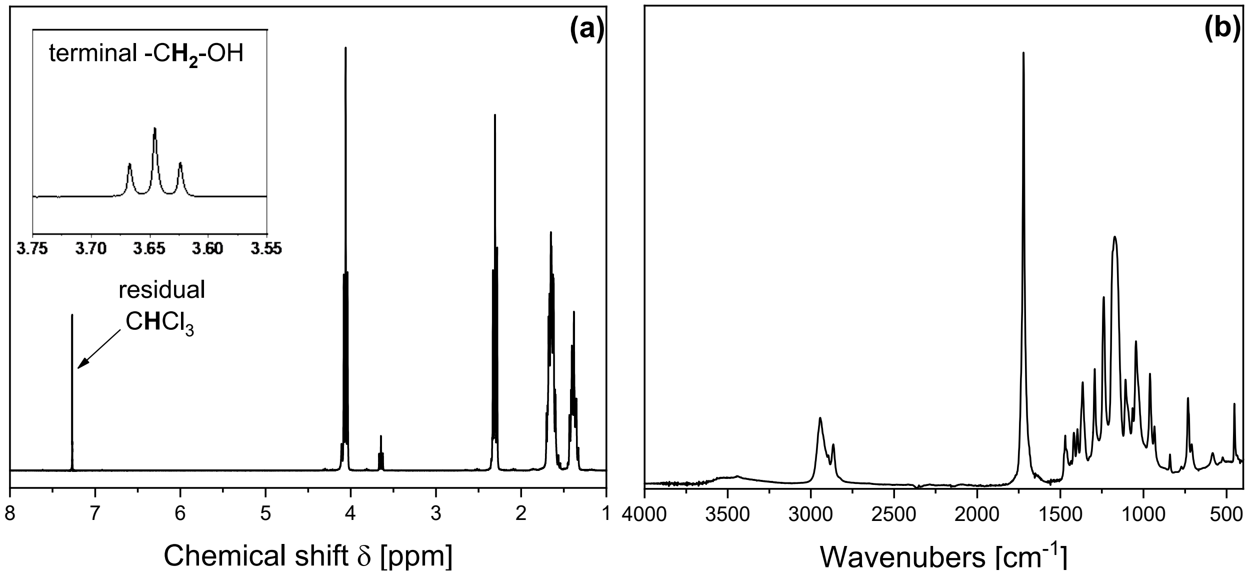

Figure S1. (a) ^{1}H-NMR spectrum and (b) FT-IR spectrum of PCL-OH.

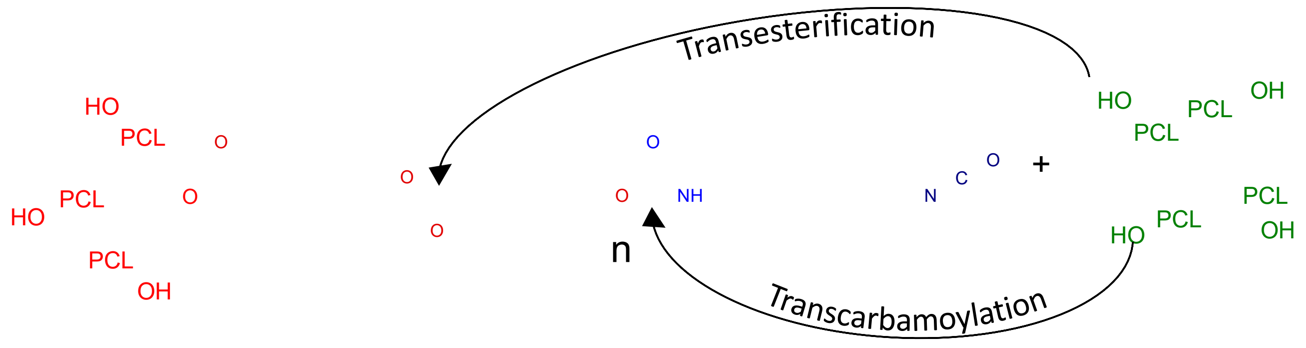

Figure S2. Detailed scheme of the possible reactions within the network.

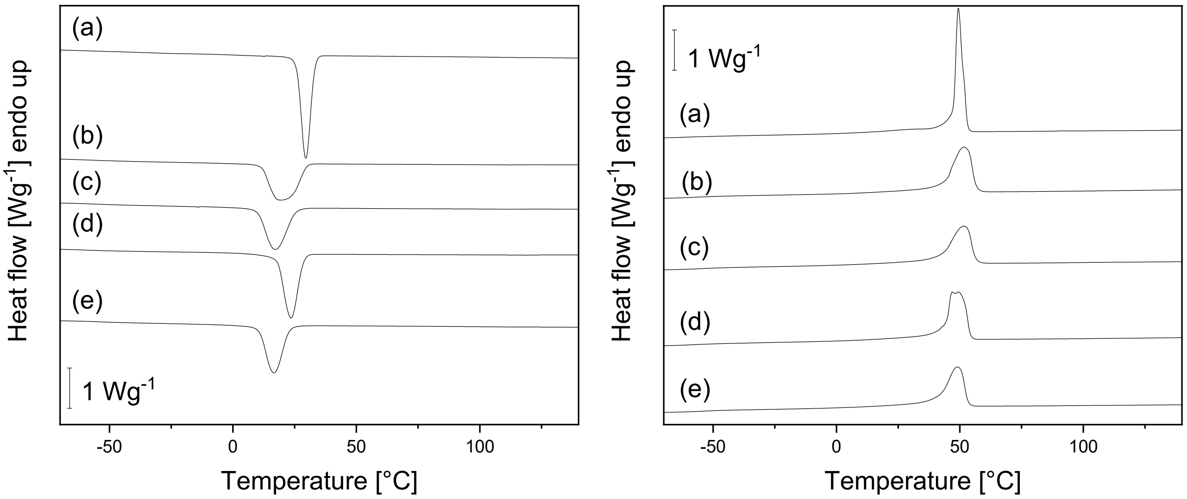

Figure S3. DSC traces, cooling (left) and second heating (right) of: (a) PCL-OH, (b) PCL-OH_MDI_1:1_120, (c) PCL-OH_MDI_1:1.33_120, (d) PCL-OH_MDI_1:1_200 and (e) PCL-OH_MDI_1:1.33_200.

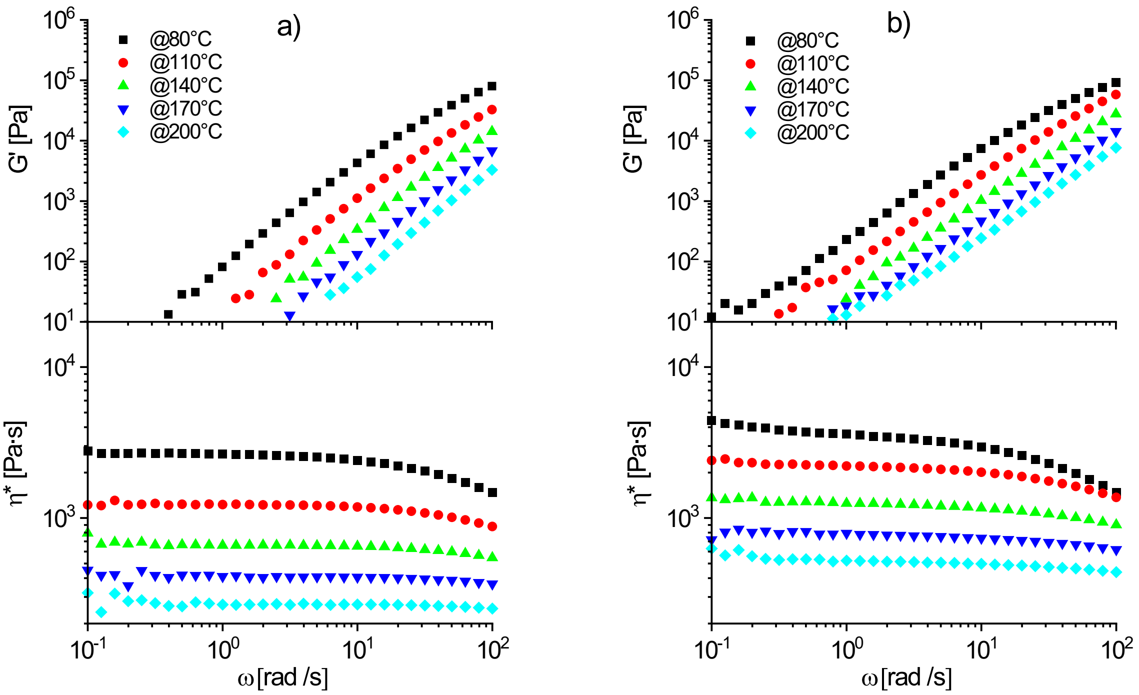

Figure S4. Rheological analyses plots, *i.e.*, storage modulus (G′) and complex viscosity (η*) for (a) PCL and (b) PCL-G.

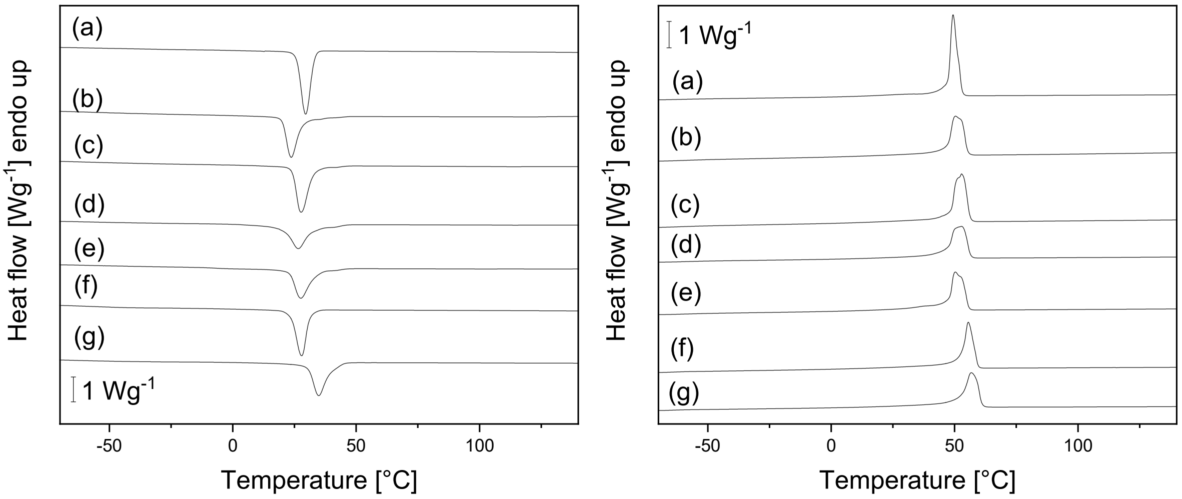

Figure S5. DSC traces, cooling (left) and second heating (right) of: (a) PCL-OH, (b) PCL-OH_MDI_1:1_120_G, (c) PCL-OH_MDI_1:1.33_120_G, (d) PCL-OH_MDI_1:1_200_G, (e) PCL-OH_MDI_1:1.33_200_G, (f) PCL and (g) PCL_G.
